# Electromagnetic field-enhanced novel tubular electrocoagulation cell for effective and low-cost color removal of beet sugar industry wastewater

**DOI:** 10.1038/s41598-023-35182-9

**Published:** 2023-05-29

**Authors:** Olfat A. Fadali, Rasha H. Ali, Mamdouh M. Nassar, Mohamed S. Mahmoud, Marwa M. Abdel-Aty, Nasser A. M. Barakat

**Affiliations:** 1 Faculty of Engineering, Chemical Engineering Department, Minia University, El-Minia, 61516 Egypt; 2 Department of Engineering, University of Technology and Applied Sciences, 311 Suhar, Oman

**Keywords:** Environmental sciences, Engineering

## Abstract

The treatment of real beet sugar mill effluent by a modified electrocoagulation process is proposed. An innovative design of an electromagnetic field-enhanced electrochemical cell consisting of a tubular screen roll anode and two cathodes (an inner and outer cathode) has been used. Different parameters have been investigated including current density, effluent concentration, NaCl concentration, rpm, number of screen layers per anode, and the effect of addition and direction of an electromagnetic field. The results showed that, under the optimum conditions, current density of 3.13 A/m^2^, two screens per anode, NaCl concentration of 12 g/l, and rotation speed of 120 rpm, the percentage of color removal was 85.5% and the electrical energy consumption was 3.595 kWh/m^3^. However, the presence of an electromagnetic field distinctly enhanced the energy consumption and the color removal percentage. Numerically, applying the magnetic field resulted in performing a color removal efficiency of 97.7% using a power consumption of 2.569 KWh/m^3^ which is considered a distinct achievement in industrial wastewater treatment process. The strong enhancement in color removal using a low power consumption significantly reduced the required treatment cost; the estimated treatment cost was 0.00017 $/h.m^2^. This design has proven to be a promising one for the continuous treatment of beet sugar industrial effluents and to be a competitor to the currently available techniques.

## Introduction

The sugar industry is one of the most water-intensive processes that produces a huge amount of heavily polluted wastewater. In the modern beet sugar industry, about 1.5^3 ^of water is consumed and about 0.5 m^3^ is discharged per metric ton of beet sugar by operating in a nearly closed circuit^1^. The discharged wastewater is characterized by high organic loads and intense color. The typical level of BOD_5_ in beet wastewater is in the range of 4000–7000 mg/L while the COD can reach from 8000 to 10,000 mg/l^2,3^. In addition to organic matter and color, the wastewater from the beet industry contains crop pests, pesticides and pathogens. The color of the effluent ranges between pale yellow and brown^4,5^. Coloring materials are soluble compounds and represent one of the most hazardous environmental pollutants in the sugar industry. The colored compounds are polymers with different molecular weights, structures, and properties. These compounds are formed in the process because of sugar degradation. Coca et al^6^ reported that the color in these wastewaters is produced mainly by two groups: melanoidin and caramels. The composition of melanoids depends on the reaction conditions; mainly temperature, heating time, pH, and nature of reactants^6,7^. Pant, D. and A. Adholeya^7^ suggested the following empirical formula of melanoidin: C_17–18_ H_26–29_ O_10_ N. Also, according to Davis^8^, caramels are formed by controlled thermal degradation of beet sugar (sucrose). They are formed due to the heating of beet sugar syrup at a high temperature and pH from 3 to 9. By caramelization of sucrose, three main product groups are responsible for the brown color; a dehydration product, caramelans (C_12_H_18_O_9_), and two polymers (caramelen (C_36_H_50_O_25_) and caramelins (C_125_H_188_O_80_)).

Overall, the advanced industrial wastewater treatment processes may contain adsorption, photo-degradation, electrochemical oxidation, Fenton oxidation, ion exchange, and biological and membrane separation^9^. The electrochemical techniques, such as electrochemical oxidation, electrochemical coagulation and electrochemical floatation, are widely used to treat heavily polluted colored organic effluents^10–12^ including sugar mill effluents^13–17^. In contrast to the conventional coagulation processes, electrocoagulation (EC) has the merit of generating coagulants locally. Aluminum and iron are exclusively used as anode materials in the EC process.

Specifically, for the sugar industry wastewaters, the electrochemical coagulation draws the most attention due to the high efficiency. For instance, approximately 60–70% of COD reduction values are reported for the sugar industry wastewater from initial COD content of 6000 mg/l^18^. However, the treatment cost stills high which limits the wide applications^2,19–21^. In this regard, several trials have been proposed to decrease the cost and maintain the high removal efficiency. For instance, some researchers have focused on the design of the cell constituents^19,22^. And others focused on the mode of flow of the wastewater^23^ and the integration of EC with other treatment processes^24,25^. However, more efforts are needed to make this effective treatment process an economically recommended methodology.

Compared to iron, the aluminum anode has disadvantages constraining its wide application on the industrial scale. For instance, the formation of a passive layer requires the addition of a relatively high amount of chlorine ion (e.g. NaCl)^26^. Conductivity increase (especially by NaCl addition) during the treatment of drinking water is highly limited in accordance with the standard norms that define the maximum chloride concentration in industrial effluent at 252 mg/L^27^. Moreover, in comparison to aluminum, iron has two important advantages. First, it is non-toxic, thus it may be used to make drinkable water, even if the Moroccan standard is 200 ppb for organoleptic and aesthetic reasons the same as aluminum. The second is that iron costs less per kilogram than aluminum (Fe costs between 0.5 and 0.8 $/kg, compared to 1.5–3 $/kg for Al). However, the main drawbacks of utilizing Fe anodes in the EC are the weak buffering effect of Fe, compared to Al, and the high solubility of Fe^2^^+^ ions. Typically, the Lewis acidity of aluminum counterbalances the generation of OH^−^ anions at the cathode, causing a buffer effect and resulting in a final pH between 7 and 8^28^. On the other hand, the buffer effect reported for iron is weaker than for aluminum; a final pH usually achieved is 9 or 10 with Fe electrodes even when the initial pH is acidic^29,30^. The high solubility Fe^2^^+^ negatively affects the formation of the efficient colloid destabilization by Fe(OH)_3_, hereby causing poor EC performance^31^. Practically, different trials are going on to enhance the performance of the Fe-EC treatment process to properly exploit this abundant and cheap metal in the treatment of industrial wastewater.

In this regard, several strategies have been introduced to enhance the performance of the Fe-EC process including aerating the water to increase the dissolved oxygen concentration and Fe^2^^+^ oxidation, increasing the pH to 7.5 or higher to improve the Fe^2^^+^ oxidation rate, introduce an alternating oxidant such and increasing the residence time to achieve complete Fe^2^^+^ oxidation^29,32,33^. Moreover, the EC cell geometry and electrode design considerably influence the treatment performance^34,35^.

Magnetohydrodynamics (MHD) is the field incorporating the magnetic field and electrically conducted fluid systems^36^. The main point of this field is that the electrically conductive fluids can also be induced by the application of a magnetic field. Additional forces will be generated in the presence of magnetic fields like the Lorentz force, the paramagnetic force due to the presence of paramagnetic ions, and the magnetic gradient force. These forces will contribute to altering the arbitrary movements of ions within the electrolyte solution. Although Coey and Hinds claimed that the electromagnetic field (EMF) alone is much smaller than other forces acting in the electrochemical reaction (like diffusion and migration), they concluded that the effect of the Lorenz force imposed by the EMF should not be neglected in the event that it is combined with another convictive movement in the electrolyte^37^. The utilization of EMF in chemical reactions has been studied intensively^38–40^. The main investigation was directed toward plasma reactions and fluid flow through pipes.

In this study, the effect of cell geometrical parameters and the addition of EMF for the treatment of real beet sugar effluent by EC process are investigated. This work was directed to use a new electrochemical cell that consists of a tubular screen array anode and two cathodes (an inner and outer cathode). The parameters that were studied are the current density, electrolyte concentration, NaCl concentration, electrolyte stirring, number of electrode pairs, and application of electromagnetic field. The novelty of this work lies in the design of the electrochemical cell and the utilization of EMF to reduce color removal time. Our results showed a high color removal efficiency with small power consumption and highly accepted required cost.

## Experimental

### Electrochemical cell setup

**Figure 1:**
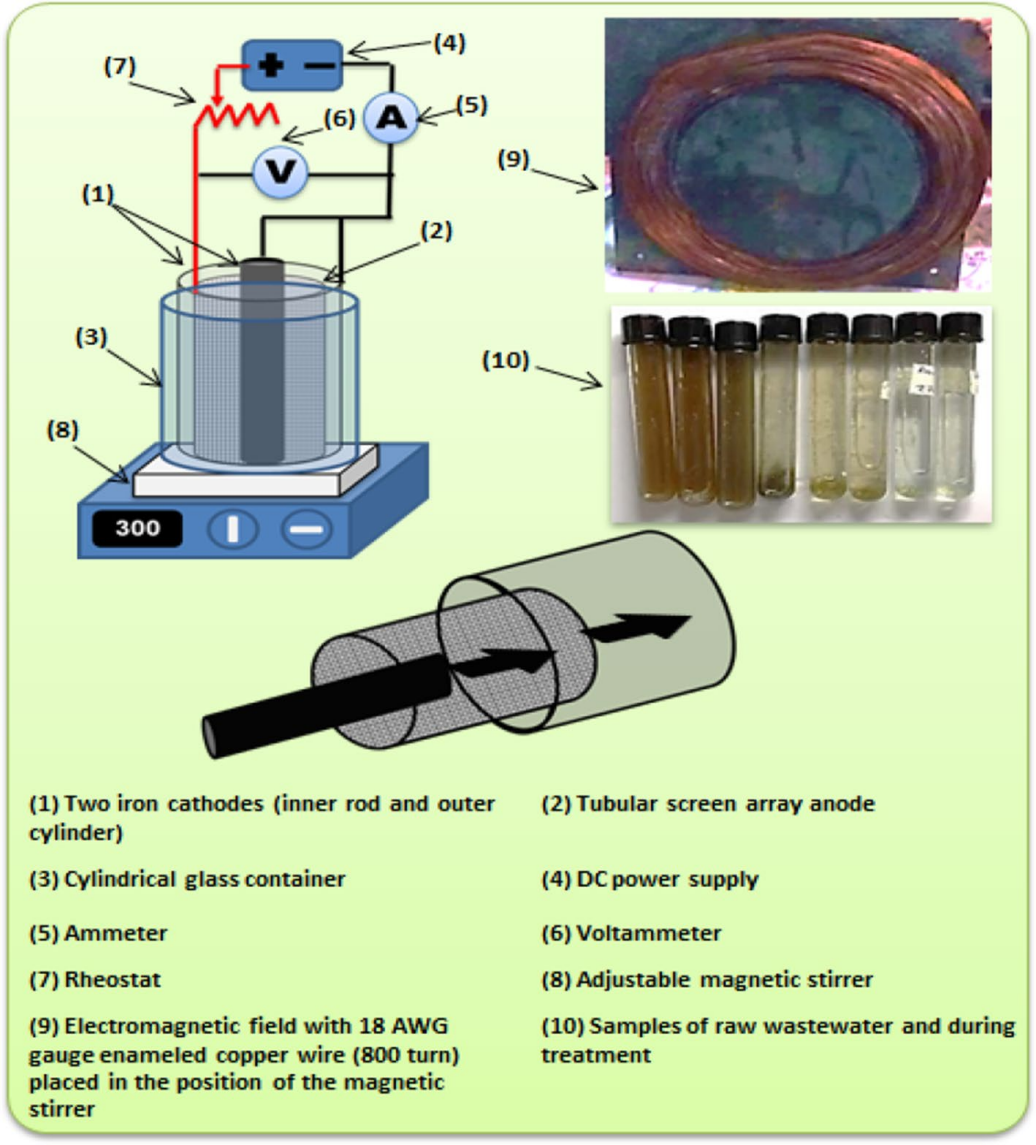
Experimental setup for the electrochemical cell, electrodes, samples, and the electromagnetic field.

The cell used in the present study consists of a tubular array of closely spaced steel screens as anode and two cathodes. These two cathodes are the inner steel rod and the outer cylindrical sheet surrounding the screen anode. Electrodes were placed in a glass container of 0.09 m in diameter and 0.125 m in height. Low carbon steel woven wire of 32 meshes has been used as an anode. It has a height of 0.04 m and an average diameter of 0.038 m. The outer cathode has a height of 0.05 m. and a diameter of 0.058 m, while the inner cathode rod has a height of 0.05 m and 0.019 m in diameter. The inner gap between the electrodes was maintained at 0.01 m. All electrodes are submerged in real wastewater from Abu-Qurqas sugar factory (Abu Qurqas City, Minia Governorate, Egypt). The cathodes and anode were connected to the 16 V DC power supply (ADAK- PS 808) by a copper wire. As shown in Fig. 1, ammeters and voltammeters were used to measure current and cell potential. A magnetic stirrer was used to rotate the wastewater solution to overcome the diffusion resistance of the ions in the vicinity of the anode and the cathodes. In another set of experiments, an electromagnetic solenoid was inserted below the electrochemical cell. The solenoid consists of an iron core with a 0.13 m diameter, a 0.001 m height, and a relative permeability of *k* = 100. Surrounding the core, an 18 AWG gauge enameled copper wire was wounded with N = 800 turns as shown in Fig. 1. The magnetic field was connected to the DC power supply and the intensity was calculated by:

1 $$B=\mu ni$$

where *μ* is the permeability (*μ* = *μ*_*o*_ × *k*), *i* is the current and n is the turn density (*n* = *N*/*L*, N is the number of turns and L is the solenoid length).

### Experimental procedures

Before charging the wastewater (0.25 L) to the cell, the conductivity was adjusted by adding NaCl solution at a specified concentration (from 6 to 30 g/L), NaCl supplied by Al Safa company, Egypt. Samples (0.003 L) were withdrawn at different time intervals and centrifuged, then analyzed for color removal measurement, using a UV spectrophotometer (Shimadzu model, UV 1601) at a wavelength of 274 nm. The variables studied are effluent concentration, NaCl concentration, current density (CD), stirring speed, number of anode screens (1- 3 screens) and effect of EMF; magnitude and direction. Typical characteristics of the used wastewater are shown in Table 1.

**Table 1:** Characteristics of the wastewater obtained from beet sugar distillation unit at Abu Qurqas sugar factory, Egypt.

pH	COD (mg/L)	Na^+^ (mg/L)	Ca^2^^+^ (mg/L)	Total P (mg/L)	SO_4_^2−^ (mg/L)	K^+^ (mg/L)	Conductivity (μS/cm)	TDS (mg/L)	Turbidity NTU
6.9	3960	355.28	33.8	17.13	28	350.4	3580	1760	0.287

### Energy consumption calculation

The energy consumption for treatment of 1 m^3^ of sugar effluent was calculated from the voltage and current readings, and the treatment time according to the following equation^41^.

2 $$E=\frac{IVt}{1000*\mathcal{V}}$$

where E is the energy consumption (kWh/m^3^), *V* is the electrocoagulation cell voltage (volt), *I* is the current intensity (A), $\mathcal{V}$ is the treated volume in m^3^, and *t* is the treatment time (h). The energy consumed to remove the organic pollutants from sugar effluent using EC coupled with EMF was calculated using Eq. (3).

3 $$E=\frac{\left({\left(IV\right)}_{electrocoagnulation}+{\left(IV\right)}_{magnetic}\right)*t}{1000*\mathcal{V}}$$

## Results and discussion

### Effect of current density

**Figure 2:**
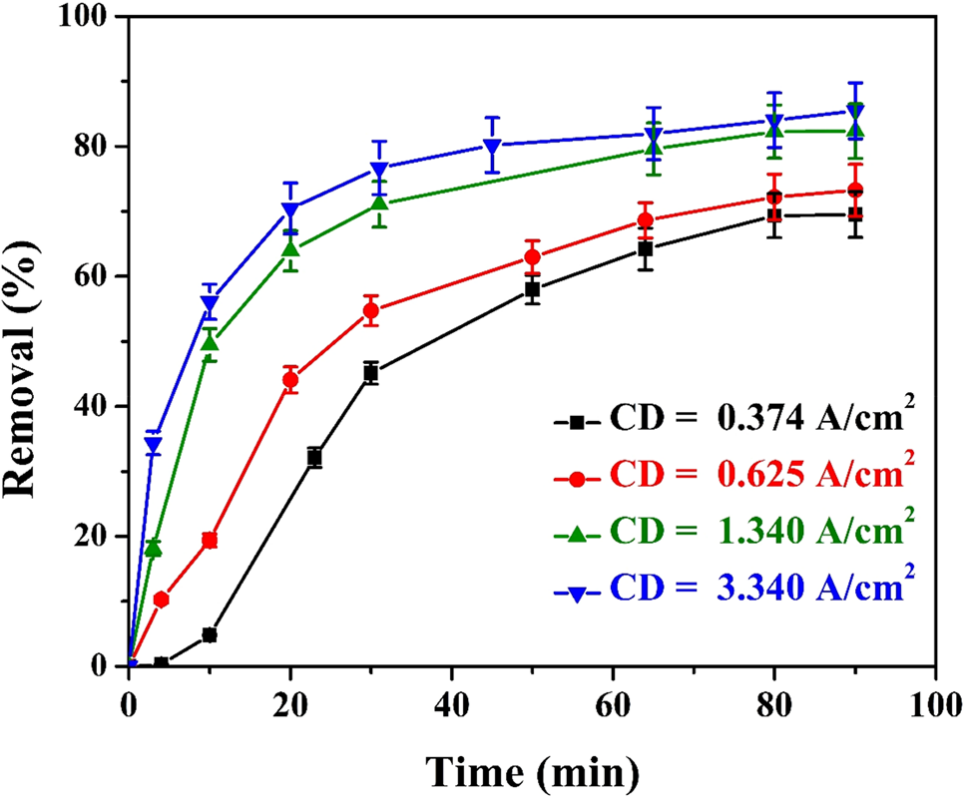
Effect of changing the CD on the percentage of color removal under the following constant conditions: ([NaCl] = 12 g/L, no. of anode screen = 1, electrolyte concentration = 100% of the raw wastewater, rpm = 0, and B = 0 Tesla).

Figure 2 shows the relationship between the color removal percentage from the utilized wastewater with time at different current densities (CD). During electrolysis, the anodic electro-dissolution leads to the release of coagulant species. The color removal depends directly on the concentration of metal ions produced by the anode. Increasing the reaction time increases the concentration of metal ions dispatched from the anode surface. Consequently, more iron hydroxide will be formed in the electrolyte, which facilitates the generation and accumulation of the flocs. It is also noticeable from Fig. 2 that increasing the treatment time increases the percentage of color removal up to 85.5% at a CD 3.13 A/m^2^. Furthermore, increasing the CD from 0.374 to 3.13 A/m^2^ increases the color removal efficiency from 70 to 85.5%. The Fe^3^^+^ ions produced at the anode increase by increasing the current density according to Faraday’s law Eq. (4)^42^.

4 $$m=\frac{Q\times \mathrm{Mwt}}{ZF}$$

where *m* is mass of dispatched ions (g), Q is the total electric charge passed (C), Mwt is the atomic mass of iron, F is the Faraday constant (96,485 C /mol), and Z is the valence number of iron. Therefore, it is expected that higher current density instantaneously generates a significant amount of iron ions that react with hydroxyl ions to form local coagulant sites to initiate the formation of flocs, which in turn adsorb more of the coloring materials and promote the efficiency of color removal^43^.

During the EC process, the generated Fe(OH)_2_ molecule attracts the organic matter by surface complexation or electrostatic attraction. Thereby, imposing the organic molecules to agglomerate^44^. The mechanism of such a process starts at the anodic sites where iron ions are generated by applying an external electrical current. Then, iron cations diffuse from the surface of the anode through both electrical double layer and diffusion layer to the bulk of the electrolyte. Afterward, iron hydroxide combines with the organic pollutant by the previously mentioned mechanisms to form one floc. The flocs tend to form denser clusters. At the cathode, hydrogen gas is generated, hydrogen bubbles attach to the generated flocs and drag them to the surface.

It is also probable that the anionic color substance molecules migrate to the anode region by electrophoresis where they are neutralized, attached to the iron hydroxide, and electrocoagulated in the anode vicinity. On the other side of the cell, by increasing current density, the generation of hydrogen gas bubbles increases with a consequent increase in the number of gas bubbles that pass through the electrolyte before they are dispatched to the atmosphere. Also, as the CD increases, the extra H_2_ bubbles evolved at the cathode increase the amount of solution entrained by the rising H_2_ bubbles. The entrained solution stirs the inter-electrode gap and accelerates the mixing of iron ions with the pollutants.

### Effect of effluent concentration

**Figure 3:**
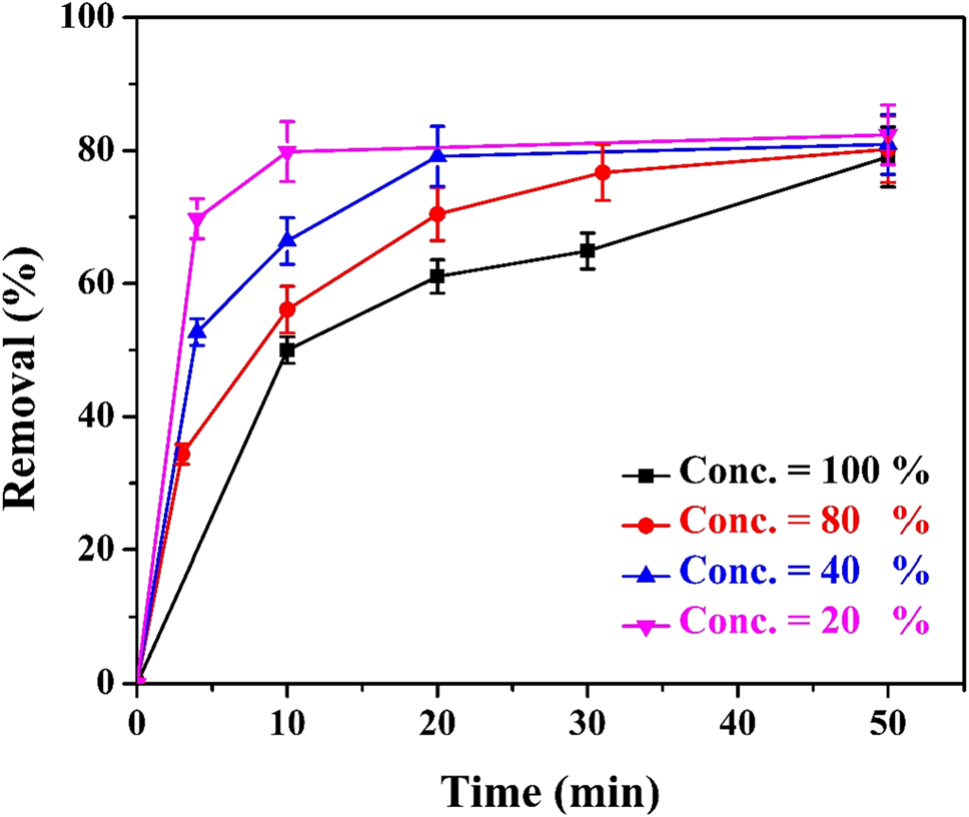
Effect of changing the effluent concentration (by dilution) on the % color removal under the following constant conditions: ([NaCl] = 12 g/l, CD = 3.13 A/m^2^, no. of anode screen = 1, rpm = 0, and B = 0 Tesla).

Influence of dilution of the used influent on the EC performance has been investigated by preparing different concentrations from the received wastewater throughout mixing with pure water. Different concentrations have been prepared; C_1_ is the effluent without dilution (100% concentration), while C_2_, C_3_, and C_4_ represent dilution percentages of 80, 40, 20%, respectively. Figure 3 shows the relationship between the % color removal with time at different initial concentrations. It is evident that, by diluting the sugar mill effluent from 100 to 20% under the same set of conditions, the percentage of color removal increased from 53 to 80%. This may be explained as follows: (1) At constant current density, the amount of Fe(OH)_3_ flocs produced is sufficient to adsorb all coloring materials in low concentrated effluent, resulting in a high percentage of color removal, whereas at high effluent concentration, the coloring materials outnumber the produced iron hydroxide flocs. (2) At high effluent concentrations, coloring molecules tend to adhere to each other forming large aggregates with low diffusion rate. This retards the rate of adsorption of coloring materials on iron hydroxide flocs, and consequently the percentage of color removal is decreased. (3) High effluent concentrations in bulk solution may also passivate the anode by increasing the amount of color molecules adsorbed on the anode surface^45^.

### Effect of electrolyte (NaCl) concentration

**Figure 4:**
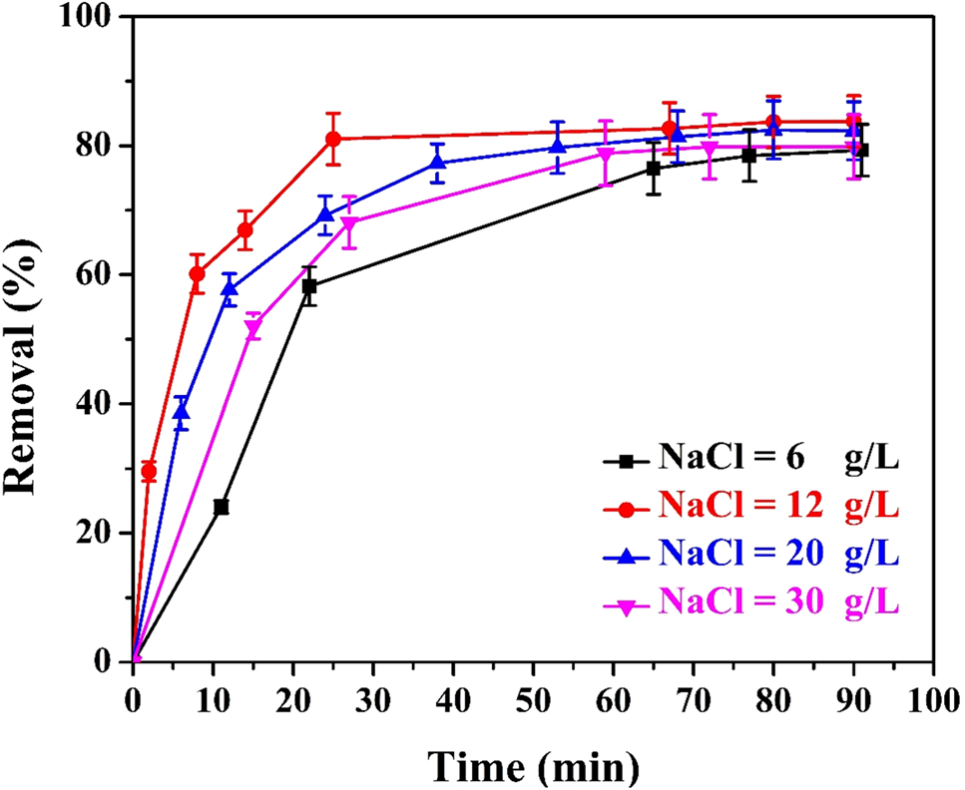
Effect of the concentration of the supporting electrolyte on the % color removal under the following constant conditions: (CD = 2.36 A/m^2^, no. of anode screen = 1, electrolyte concentration = 100% of the raw wastewater, rpm = 0, and B = 0 Tesla).

Figure 4 shows the effect of electrolyte concentration on the color removal percentage (6–30 g/l). As shown, the percentage color removal increases with increasing salt concentration until it reaches its maximum at about 12% NaCl, then decreases with further increase in NaCl. The enhancement scheme can be explained as follows: at the beginning, the increase in the percentage of color removed with increasing NaCl may be attributed to the ability of chloride ions (ionization of NaCl) to destroy passive iron oxide film formed on the iron anode during electrolysis. Thus, by sustaining the formation of Fe^2^^+^ and Fe^3^^+^ ions, it increases the amount of formed ions in the solution and improves the efficiency of color removal^46,47^. Instead, the decrease of color removal beyond 12 g NaCl/l may be attributed to the competing effect of chlorine ions’ migration to the anode surface in preference to the negatively charged colored molecule. It could be also attributed to a decrease in dissolved oxygen solubility caused by NaCl molecules salting out, which reduces the amount of Fe(OH)_3_ flocs and thus the number of sites available to adsorb color bodies.

### Effect of electrolyte stirring

**Figure 5:**
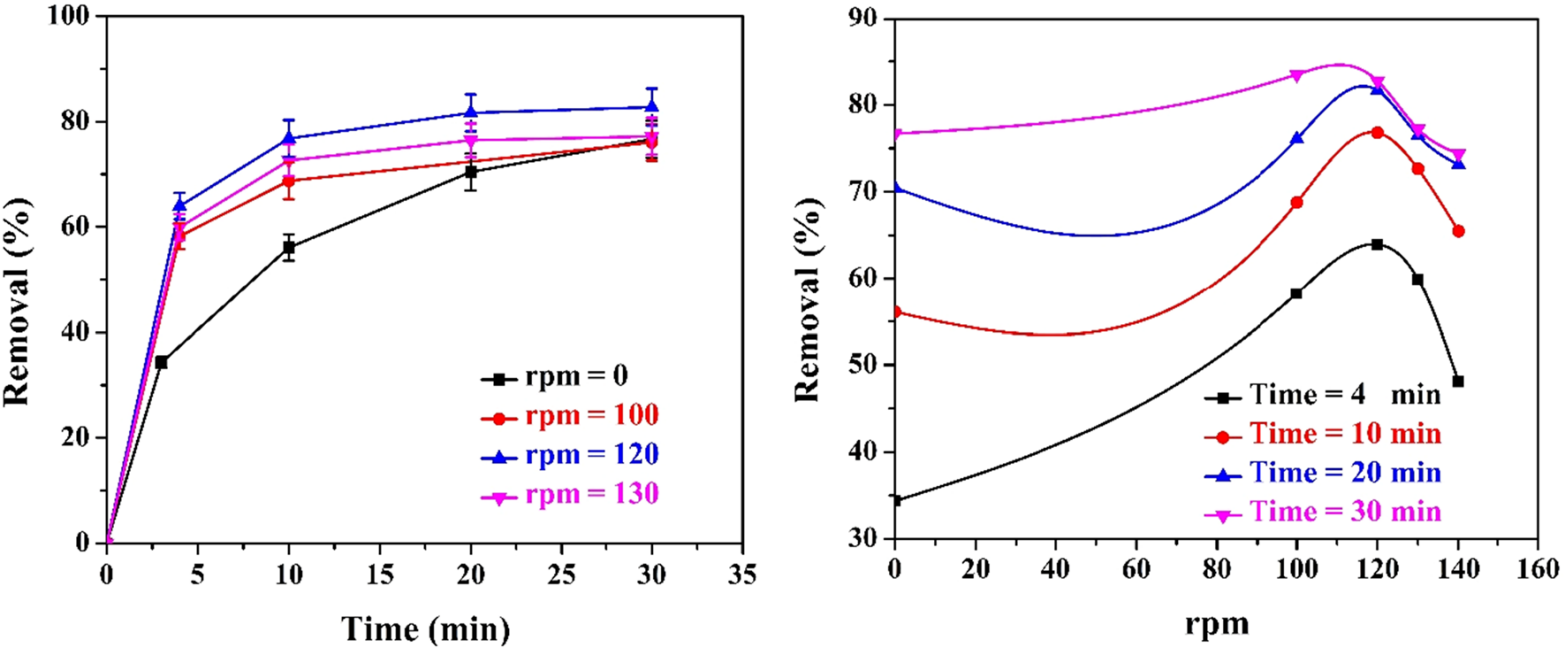
left: Effect of rotation speed (rpm) on the % color removal under the following constant conditions: (CD = 3.13 A/m^2^, no. of anode screen = 1, electrolyte concentration = 100% of the raw wastewater, and B = 0 Tesla), right: Effect of rpm on % color removal after 4–30 min.

Figure 5 shows the effect of rotational stirring speed on the wastewater color removal. The % color removal increases with the increase of rotational speed until 120 rpm, and then decreases by further increasing rpm. This can be attributed to the presence of two regimes: the first regime from 0 to 120 rpm and the other regime at a higher speed. Mild stirring improves the mixing efficiency between the color bodies and the hydrolyzed Fe^3^^+^ with a consequent increase in the percentage of color removal. Also, mild stirring may distribute H_2_ bubbles evenly in the solution, with a consequent increase in the floating ability of the flocs. On the other hand, vigorous stirring above 120 rpm disperses the coagulated color molecules in the solution with a consequent decrease in the percentage of attachment of color to iron hydroxide. This finding is in agreement with the previous work done by Khaled, B., et al. and Khalaf, A. et al. ^48,49^.

### Effect of number of anode screens

**Figure 6:**
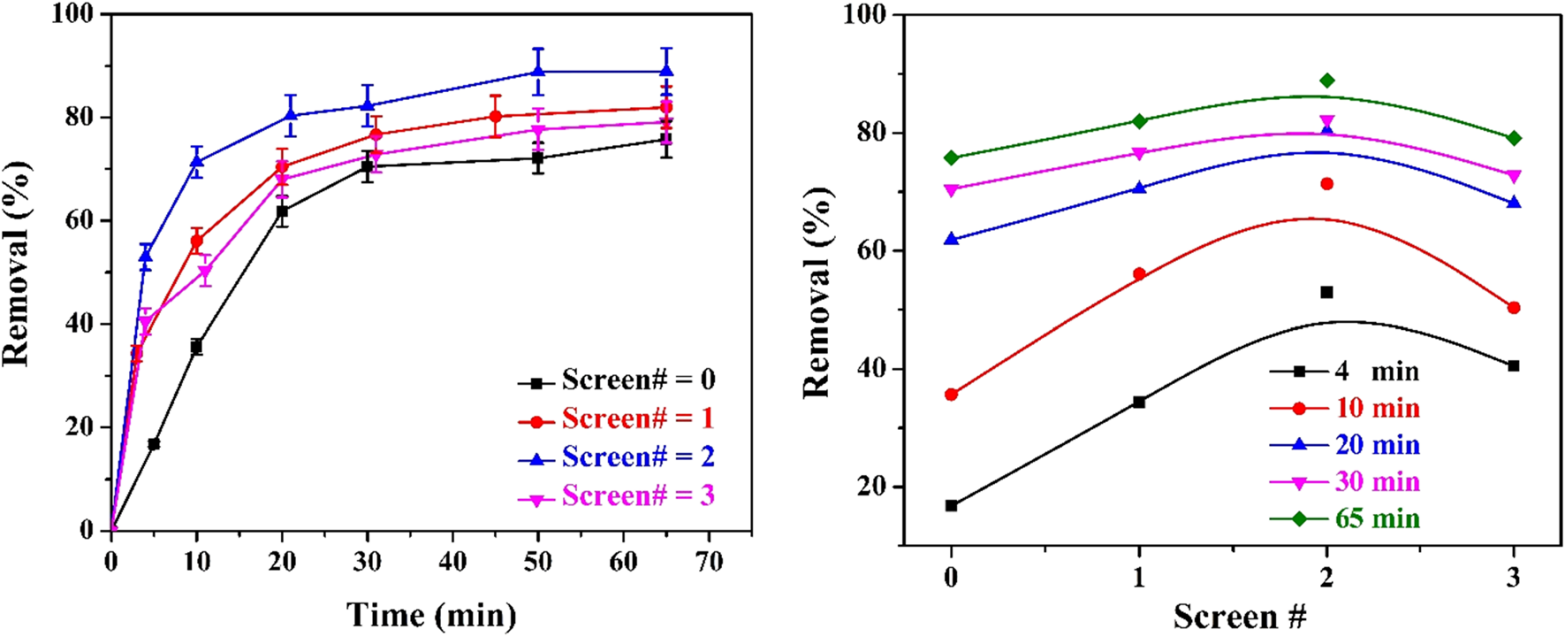
left: Effect of time on % color removal using different numbers of screen under the following constant conditions: ([NaCl] = 12 g/L, CD = 3.13 A/m^2^, electrolyte concentration = 100% of the raw wastewater, rpm = 0, and B = 0 Tesla). Right: Effect of number of screens per array anode on % color removal (zero is cylindrical sheet anode).

Extra anode screens can facilitate the iron ions’ formation inside the electrolyte. Figure 6 shows the rate of color removal using different numbers of screen anodes. The results illustrated that the percentage of color removal is higher when using double screen anodes compared to single and triple anodes. The enhancement of the rate of color removal is attributed to the fact that the porous screen anode allows free circulation of the solution entrained by the rising H_2_ bubbles, with a consequent improvement in the mixing efficiency between the anodically dissolved Fe^3^^+^ ions and the colored pollutants. The degree of enhancement had different ranges based on the time of decolorization. By the addition of a second screen, despite a decrease in current density, more opening will exist, which will function as turbulence promotors, and consequently, more turbulence will be created that will overcome the decrease in current density. This leads to more efficient hydrodynamics inside the reaction cell. However, with the addition of the third screen, the created turbulence will interfere with the existing turbulence (from the second screen) and dampen its effect as a turbulence promotor, in addition to the effect of decrease in current density, leading to a decrease in the efficiency of the system.

### Effect of addition of electromagnetic field

Since many years ago, it has been documented in several articles that the magnetic field (MF) may alter the physicochemical characteristics of water^50^. For instance, when Han et al.^51^ studied the optical characteristics of water in the presence of two powerful magnets, they discovered that the infrared absorption quality had altered. According to Holysz et al., the magnetic field can improve conductivity and reduce surface tension of the water ^52^. Using frictional studies, Wang et al. investigated the impact of a static magnetic field on the liquid water. The findings indicated that the friction coefficient was lower in presence of the magnetic field ^53^. In their study of the impact of the magnetic field on water's hydrogen bonds, Cai et al. ^54^ used molecular dynamics modeling, experimental data and theoretical models to examine the mechanism of magnetization. The results indicated that the magnetic field has a distinct impact on the hydrogen bonds. When the magnetic field varies from 0 to 900 mT, Liu et al. ^55^ reported that the magnetic field may speed up the decomposition of organic materials in pulp and paper effluent. The pH values of the wastewater initially climbed to the climax and subsequently fell. Consequently, in this study, investigation of the influence of magnetic field application on color removal efficiency has been performed.

**Figure 7:**
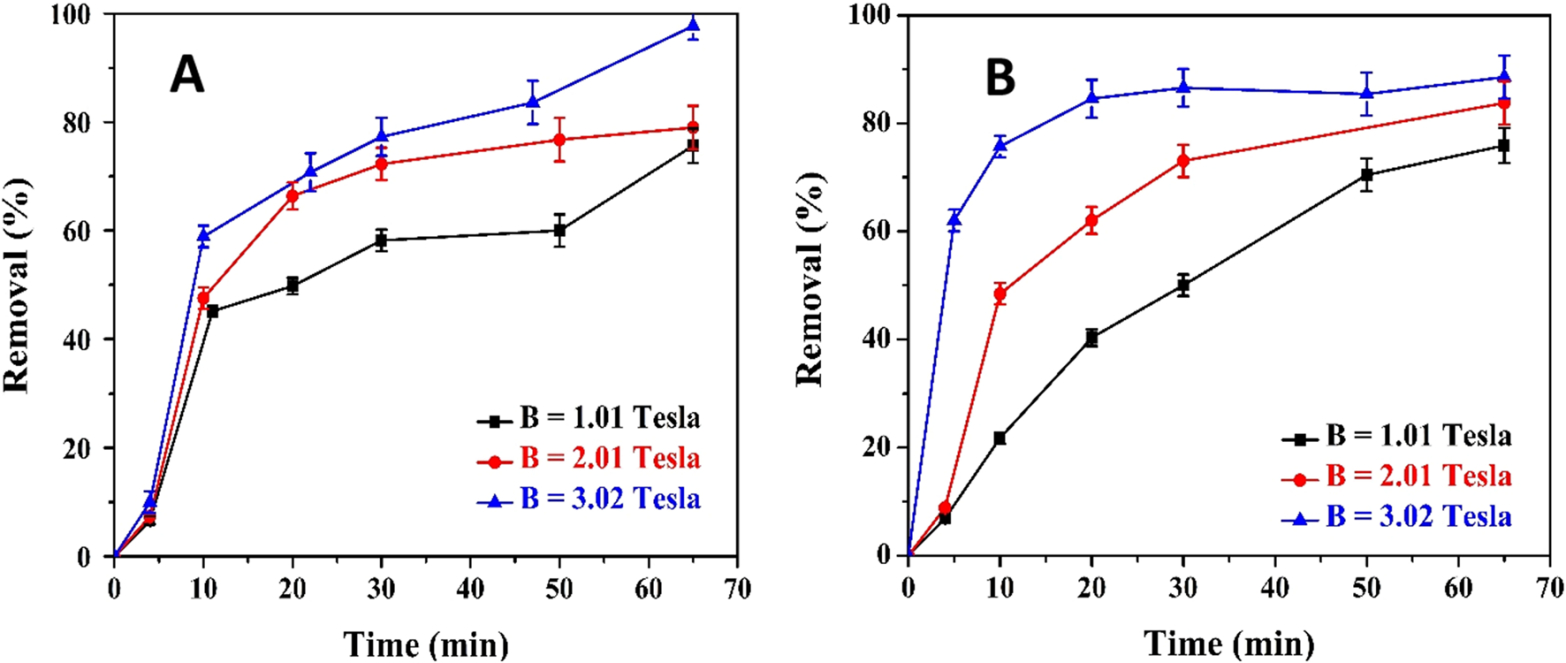
(**A**) EMF perpendicular to the electrodes, (**B**) EMF parallel to the electrodes; the experiments have been at [NaCl] = 12 g/L, CD = 3.13 A/m^2^, electrolyte concentration = 100% of the raw wastewater, rpm = 0, and two screen anodes.

Figure 7A shows the effect of the addition of EMF below the EC cell on the rate of pollutant removal. Also, Fig. 7B depicts the same behavior but with the EMF placed beside the electrodes. It is clear from both figures that the addition of the EMF enhanced the color removal percentage and rate. Moreover, increasing the magnetic field intensity promotes faster removal of color. By using B = 3.02 Tesla, the removal rate reached more than 80% in 20 min, compared with 30 min for the experiment without the EMF. It is also noticeable that the position of the EMF below (perpendicular to) the EC cell gave better enhancement of the color removal than the position beside (parallel to) the EC cell. The addition of the EMF enhanced the EC reactions due to the induced motion of the paramagnetic ions by the effect of the electric field and the additional forces imposed by the EMF in the electrolyte. Chen et al. ^56^ indicated that the value of MHD force at the electrode edge depends on the magnetic properties of the electrode materials. One of the probable actions of the MHD force at the electrode surface is to facilitate the dispatch of the hydrogen bubbles from the cathode and the iron ions from the anode. Due to the high magnetic susceptibility of the iron electrodes, the paramagnetic properties generated due to the EMF enhance the locomotion of hydrogen bubbles and make them detach from the cathode rapidly.

## Static electromagnetic field simulation

The Finite Element Method Magnetics (FEMM) software has been used to simulate the static magnetic field intensity inside the proposed electrocoagulation cell. This simulation software can deal with some low-frequency electromagnetic problems in two-dimensional planar and axisymmetric domains ^57^. In the FEMM software, the simulation setup frame typically uses Maxwell’s equations, which are expressed in terms of **E**, **B**, and **J**. Therefore, the equations are ^58^:

6 $$\nabla \cdot \mathrm{E}= \frac{\rho }{\varepsilon }$$

7 $$\nabla \times \mathrm{E}= -\frac{\partial \mathrm{B}}{\partial t}$$

8 $$\nabla \cdot \mathrm{B}= 0$$

9 $$\nabla \times \mathrm{B}= \mu \left(\mathrm{J}+\varepsilon \frac{\partial \mathrm{E}}{\partial t}\right)$$

**Figure 8:**
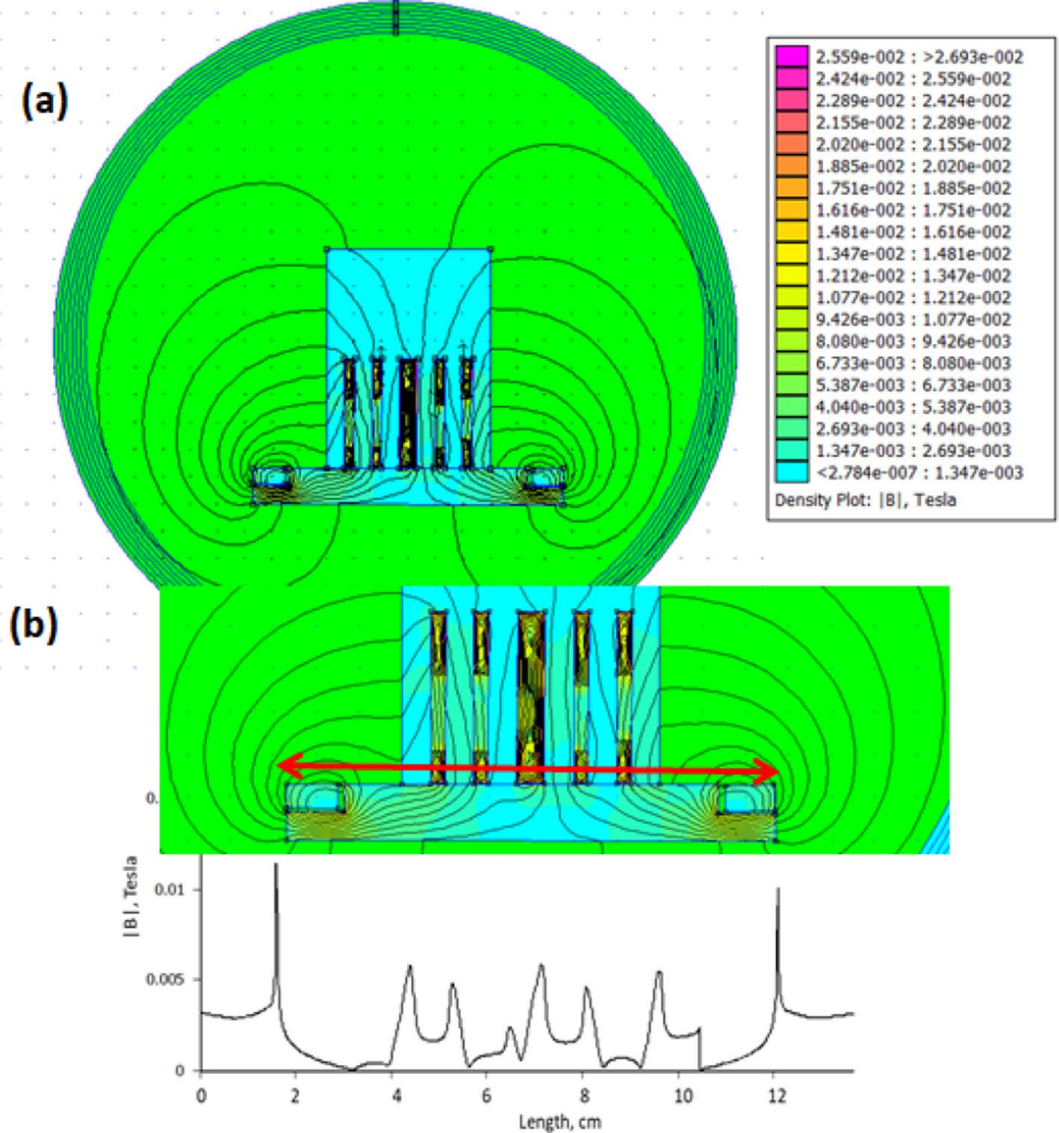
(**a**) the side view of the electromagnetic field profile inside the electrochemical cell. The simulation procedure has been conducted without the addition of the electrochemical cell potential. The color scale represents the magnetic field from 0.0 to 0.027 T. (**b**) the electromagnetic field intensity vs. linear contour (shown by the red arrow) indicating the fluctuation of intensity with the position.

Figure 8a shows the side view of the simulated 2D magnetic field profile. The contours of the magnetic field intensity of the electromagnets indicated that the magnetic flux goes out from the solenoid and passes through two pathways: i-the electrolyte due to the presence of paramagnetic ions; and ii-the steel rod, steel mesh, and the steel cylinder (electrodes). The effect in the electrolyte mainly appears between the outer cathode and the anode mesh and gradually diminishes between the inner cathode and the anode mesh. Also, the magnetic field direction is pointed downward, which will typically accelerate the settling of the generated flocs. Meanwhile, magnetic flux will also pass through the electrodes as they are ferromagnetic. This will enhance the electron movement upward through the anode or downward through the cathode. The movement of the paramagnetic ions in the electrolyte will be in a helical bath because of both electric and electromagnetic fields, and therefore, more iron ions are generated, and more reactions occur over the cathodic area. It is also noticeable that only 0.5% of the magnetic flux is directed to the electrolyte in the electrochemical cell (as shown in Fig. 8b), because the calculated flux was 3.02 Tesla while the flux inside the solution is 0.01 Tesla. There is an inconsistency between the measured and the simulated magnetic flux. This is due to the difference between the magnetic properties of those specified for simulation and those for real materials. Moreover, during the simulation, the proper magnetic properties of the electrolyte containing the paramagnetic iron ions are not fully specified. And so, the simulation is done for preliminary visualization and requires more calculations to exactly specify the dynamic behavior of the paramagnetic iron ions under both electric and magnetic fields.

## Energy consumption and economic study

**Figure 9:**
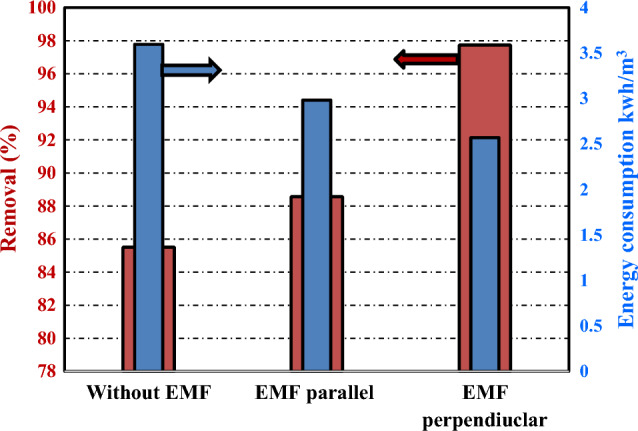
Energy consumption (in kWh/m^3^) and % color removal for the two positions of the EMF compared to the experiment without EMF.

Calculation of energy consumption using Eqs. (2 and 3) at the optimum condition (CD = 3.13 A/m^2^, NaCl concentration = 12 g/L, and two screens per anode) was found to be 3.595 kWh/m^3^ of treated wastewater. Comparison with the work done by Sahu, O. et al. ^14^ for color removal of real sugar can effluent indicated that the maximum value of energy consumption was 32.1 kWh/m^3^, which is very high compared to the current work (3.595 kWh/ m^3^). This discrepancy between the two energy consumption values may be attributed to the high efficiency of the newly proposed electrochemical cell used in the present work compared to the electrochemical reactor used by Sahu, O. et al., which consists of four pairs aluminum electrodes arranged in a parallel mono-polar mode. It is also of interest to point out that energy consumption can be further improved by the addition of EMF (from 3.595 to 2.569 kWh/m^3^) as shown in Fig. 9. This enhancement is due to the faster removal of pollutants in the presence of EMF compared to the experiment without the EMF. With an electricity tariff in Egypt of 0.075 $/kWh, the energy cost will be 0.193 $/m^3^. Table 2 summarizes the optimum color removal results and the corresponding required power using the proposed cells at different conditions.

**Table 2:** Summary for the color removal percentages along with the energy consumption using the proposed Fe-EC process.

EMF Position	% color removal	Energy consumption kWh/m^3^
Without EMF	85.51	3.59
Parallel to electrodes	88.57	2.98
Perpendicular to electrodes	97.73	2.57

For proper evaluation of the proposed treatment strategy, Table 3 shows the consumed current densities in treatment of sugar industry wastewaters using electrochemical techniques with different electrodes. The primary consideration in choosing a treatment process for any industry is its economic viability. Therefore, Table 3 also contains an analysis of the cost-effectiveness of the existing treatment process. Based on an international website (Alibaba Website on Apr. 22, 2023; 0.5, 13 and 2 $/kg for Fe, Cu and Al, respectively), the cost of the used metals plates was also estimated in Table 3. The dissolved mass of electrode (kg/h.m^2^) was calculated from Faraday’s equation (Eq. 4). As shown in the table, the required current density as well as the corresponding cost in the proposed cell is very small compared to the other reported methods. Overall, the proposed treatment strategy in this study gathers between the two main required parameters in the highly recommended treatment process, high efficiency and low cost.

**Table 3:** Current densities consumed in treatment of sugar industry waste waters using electrochemical techniques with different electrodes.

Waste	Treatment technique	Electrode design	Electrode material	Removal (%)	CD (A/m^2^)	Cost $/h.m^2^	Refs
Sugar industry	Magnetic field-enhanced Electrocoagulation	Tubular screen roll anode and two cathodes	Stainless steel electrodes	97.73% Color	3.13	0.00017	Our study
Beet sugar industry	Electrooxidation	Two parallel rectangular electrodes: one anode and one cathode	Stainless steel electrodes	75% Organics	48.5	0.034	^19^
Sugarcane processing industry	Electro-oxidation	Two copper plates were cute for electrodes preparation. The electrodes were connected in sequence	Copper	90% Color	178	3.05	^59^
Sugar industry	Electrocoagulation	Plate electrodes arranged in parallel	Iron	86% Color	178	3.19	^60^
Sugar industry	Electrocoagulation	Four plate electrodes: Two anodes and two cathodes	Aluminum electrode	88.6% Color	44.5–133.5	0.104	^61^
Simulated sugar industrial effluent	Electro-oxidation	Plate electrodes	Anode: RuO_2_-Titanium Cathode: stainless steel	80.74%COD	500	0.32	^17^
Sugar industry	Electro-oxidation	Plate electrodes	Copper	83.5% Color	178	3.28	^62^

## Conclusion

Color removal efficiency of beet sugar industry wastewater can be strongly improved by a modified design iron-based electrocoagulation cell. Using two cathodes (inner rod & outer flat sheet) and a tubular array of closely spaced screens anodes allowed uniformity of current distribution and decreased the IR drop. Also, it allowed H_2_ bubbles to stick to the pollutants efficiently. These actions distinctly improve the color removal specially when the operating parameters are optimized. The optimum NaCl electrolyte concentration is 12 g/L. Increasing number of screens above two decreases the efficiency of the process, due to hindering of solution circulation. Optimum rpm was 120 above this value, re-dispersion of the adsorbed colored pollutants takes place. Moreover, the presence of electromagnetic field reduced the time required for removal of the pollutant therefore enhanced the energy consumption. Overall, the results indicated that the proposed new cell deign and magnetic field assisting are effective parameters in term of decreasing the required current density and performing highly acceptable color removing efficiency with economically recommended treatment cost.

## Data Availability

All data generated or analyzed during this study are included in this published article.
